# Supplementary material for: Multiscale modelling shows how cell-ECM interactions impact ECM fibre alignment and cell detachment
Source: PLoS Comput Biol. 2025 Nov 26;21(11):e1012698. doi: 10.1371/journal.pcbi.1012698 (PMC12680367; doi:10.1371/journal.pcbi.1012698)
Supplement: S1 Table — Description and values of the parameters used in our simulations. (PDF) [file pcbi.1012698.s003.pdf]

Multiscale modelling shows how cell-ECM interactions impact ECM fibre alignment and cell detachment

Juan Arellano-Tintó [1,2 *], Daria Stepanova[1], Helen M. Byrne[3, 4], Philip K. Maini[3], Tomás Alarcón[5,1,2,6]

1 Centre de Recerca Matemàtica, Bellaterra (Barcelona), Spain

2 Departament de Matemàtiques, Universitat Autònoma de Barcelona, Bellaterra (Barcelona), Spain

3 Wolfson Centre for Mathematical Biology, Mathematical Institute, University of Oxford, Oxford, UK

4 Ludwig Institute for Cancer Research, Nuffield Department of Medicine, University of Oxford, Oxford, UK

5 Institució Catalana de Recerca i Estudis Avançats (ICREA), Barcelona, Spain

6 Barcelona Collaboratorium for Theoretical & Predictive Biology, Barcelona, Spain

* jarellano@crm.cat

Table 1. Model parameters. Description and values of the parameters used in our simulations.

**General parameters**	
$\eta = 50$ pN·s·μm^{-1}	Viscous coefficient. Estimated
**ECM parameters**	
$L^0_{l,m}$	Natural length of the fibre.<br>Determined by the initial condition
$k^{\text{el}}_{l,m} = \frac{E_{l,m}A_{l,m}}{L^0_{l,m}}$	Elastic constant of the fibre
$E_{l,m}$<br>$E_{l,m} = E_{\text{t}} = 10 - 100$ MPa<br>$E_{l,m} = E_{\text{c}} = 0.1E_{\text{t}}$ MPa	Young's modulus of the fibres. Reference value from [1, 2]<br>Young's modulus of extended fibres<br>Young's modulus of compressed fibres [2]
$A_{l,m}$<br>Fibre diameter $= 10^{-7}$ m	Cross-sectional area of the fibres<br>[3, 4]
Crosslink density, $0.2\,\mu\text{m}^{-2}$	[2]
Mean fibre length density, $0.6 - 0.7\,\mu\text{m} \cdot \mu\text{m}^{-2}$	[2]
**Cell parameters**	
$\kappa^{\text{el}}_i = 5 \cdot 10^3$ pN·μm^{-1}	Elastic constant of cell viscoelastic elements. Estimated
$\kappa^{\text{vi}}_i = 10^3$ pN·s·μm^{-1}	Damping constant of cell viscoelastic elements. Estimated
$l^{\text{c}}_i = 15$ μm	Natural length of viscoelastic elements.<br>Estimated from cell sizes.
$\kappa^{\text{el}}_i = 1$ pN·rad^{-1}	Angular elastic constant of the viscoelastic elements.<br>Estimated
$\theta^0_{i,j}$	Natural angle between viscoelastic elements.<br>Determined by initial cell shape.
$F^{\text{co}} = 10^3 - 5 \times 10^4$ pN	Estimated. Reference values [5–7].
**Integrin and FAs parameters**	
$k^{\text{i}} = 10^6$pN $\cdot\,\mu$m^{-1}	Elastic constant of the integrins, see [8].
$\text{K}_{\text{on}} = 0.2\,\text{s}^{-1}$	Integrins binding rate, estimated value.<br>Reference value for estimation [7, 9]
$\text{K}_{\text{off}}(F^{\text{i}}_{i,l}) = 0.4 \exp\left(-0.04F^{\text{i}}_{i,l}\right) + 4 \times 10^{-7} \exp\left(0.2F^{\text{i}}_{i,l}\right)$	Integrin unbinding rate, [9]
$N = \frac{6000}{N^{\text{c}}_n}$ integrins	Estimated available integrins per FA site.<br>N^{c}_n is the number of FA sites in the n-th cell

References

1. Tsingos E, Bakker BH, Keijzer KA, Hupkes HJ, Merks RM. Hybrid cellular Potts and bead-spring modeling of cells in fibrous extracellular matrix. Biophysical Journal. 2023;122(13):2609–2622.

2. Humphries D, Grogan J, Gaffney E. Mechanical cell–cell communication in fibrous networks: the importance of network geometry. Bulletin of Mathematical Biology. 2017;79:498–524.

3. Lindström SB, Vader DA, Kulachenko A, Weitz DA. Biopolymer network geometries: Characterization, regeneration, and elastic properties. Physical Review E. 2010;82(5):051905.

4. Liang L, Jones C, Chen S, Sun B, Jiao Y. Heterogeneous force network in 3D cellularized collagen networks. Physical Biology. 2016;13(6):066001.

5. Elosegui-Artola A, Oria R, Chen Y, Kosmalska A, Pérez-González C, Castro N, et al. Mechanical regulation of a molecular clutch defines force transmission and transduction in response to matrix rigidity. Nature cell biology. 2016;18(5):540–548.

6. Novikova EA, Storm C. Contractile fibers and catch-bond clusters: a biological force sensor? Biophysical journal. 2013;105(6):1336–1345.

7. Sabass B, Schwarz US. Modeling cytoskeletal flow over adhesion sites: competition between stochastic bond dynamics and intracellular relaxation. Journal of Physics: Condensed Matter. 2010;22(19):194112.

8. Lepzelter D, Bates O, Zaman M. Integrin clustering in two and three dimensions. Langmuir. 2012;28(12):5379–5386.

9. Li Y, Bhimalapuram P, Dinner AR. Model for how retrograde actin flow regulates adhesion traction stresses. Journal of Physics: Condensed Matter. 2010;22(19):194113.
